# Chronically infused angiotensin II induces depressive-like behavior via microglia activation

**DOI:** 10.1038/s41598-020-79096-2

**Published:** 2020-12-16

**Authors:** Hyun-Sun Park, Min-Jung You, Bohyun Yang, Kyu Beom Jang, Jongman Yoo, Hyun Jin Choi, Sang-Hyuk Lee, Minji Bang, Min-Soo Kwon

**Affiliations:** 1 Department of Pharmacology, Research Institute for Basic Medical Science, School of Medicine, CHA University, CHABIOCOMPLEX, 335 Pangyo, Bundang-gu, Seongnam-si, Gyeonggi-do 13488 Republic of Korea; 2 Department of Microbiology and School of Medicine, CHA University, CHABIOCOMPLEX, 335 Pangyo, Bundang-gu, Seongnam-si, Gyeonggi-do 13488 Republic of Korea; 3 College of Pharmacy and Institute of Pharmaceutical Sciences, CHA University, CHABIOCOMPLEX, 335 Pangyo, Bundang-gu, Seongnam-si, Gyeonggi-do 13488 Republic of Korea; 4 Department of Psychiatry, CHA Bundang Medical Center, CHA University, 59 Yatap-ro, Bundang-gu, Seongnam-si, Gyeonggi-do 13496 Republic of Korea

**Keywords:** Depression, Molecular neuroscience

## Abstract

Brain inflammation is one of hypotheses explaining complex pathomechanisms of depression. Angiotensin II (ANGII), which is associated with hypertension, also induces brain inflammation. However, there is no animal study showing the direct relationship between ANGII and depression. To address this issue, ANGII-containing osmotic pumps were implanted into adult male C57BL/6 mice subcutaneously for subacute (7 days) and chronic (at least 21 days) periods and behavioral and molecular analyses were conducted. Chronic infusion of ANGII into mice induced depressive-like behaviors, including the tail suspension test and forced swimming test, which were reversed by imipramine. Chronic infusion of ANGII also induced microglial activation in the hippocampus with increase of Il-1β mRNA and decrease of Arg1 mRNA. In addition, chronic ANGII infusion activated the hypothalamic–pituitary–adrenal axis (HPA axis) and resulted in decreased hippocampal glucocorticoid receptor level. However, subacute ANGII infusion did not induce significant molecular and behavioral changes in mice compared to that of control. The molecular and behavioral changes by chronic ANGII infusion were reversed by co-treatment of minocycline or telmisartan. In addition, ANGII treatment also induced the pro-inflammatory changes in BV-2 microglial cells. Our results indicate that ANGII can induce depressive-like behaviors via microglial activation in the hippocampus and HPA axis hyperactivation in mice. These might suggest possible mechanism on depressive symptom in chronic hypertensive state.

## Introduction

Researchers have tried to establish a relationship between medical illness and mental disorders. People with a chronic somatic disease like cancer, heart disease, and hypertension are relatively more at risk of psychological distress than are healthy people^1^. Although the direct correlation is controversial^2–4^, hypertension seemed to be closely related to depression. Men with cardiovascular disease, including hypertension, were prone to depressive symptoms, and consequently, depression and general anxiety disorder^5,6^. Depression can be a risk factor for cardiovascular disease development, which in turn is associated with hypertension^7–9^. Depression increases the incidence of hypertension^10^, and clinical symptoms of depression were reported to be associated with elevated blood pressure in elderly adults and women^11,12^.

In healthy people, the renin–angiotensin–aldosterone system (RAAS) is important to maintain homeostasis in blood pressure control. Angiotensin II (ANGII), synthesized from angiotensinogen through the sequential actions of renin and angiotensin converting enzyme (ACE), exhibits physiologic actions via ANGII receptors, such as AT_1_R and AT_2_R^13^. ANGII is known to promote hypertensive state by enhancing sympathetic outflow, altering the release of hormones associated with modulating body fluid balance via AT1R actions in heart, kidney, blood vessels, adrenal glands, and cardiovascular control centers in the brain^14,15^.

In addition to hemodynamic effect, ANGII also have non-hemodynamic effect including pro-inflammatory effect^16^. ANGII showed pro-inflammatory effect via activating pro-inflammatory transcription factor such as NF-κB and toll-like receptor 4 pathway and stimulating lymphocytes directly in vitro^17^. Further, peripheral ANGII can affect the brain and even cause inflammation in the brain such as the hippocampus in vivo^18–20^. ANGII receptor blocker (ARB) ameliorated brain inflammation^21^ and telmisartan (an AT_1_R blocker) reduced glial activation in vivo and in vitro^22^.

Considered that brain inflammation can attribute to depression, ANGII seemed to be associated with depression. There are various hypotheses explaining depression and among them, the most well-known classic hypothesis explaining depression is monoamine hypothesis which posits that depression is caused by decreased monoamine (e.g. serotonin, noradrenaline) function in the brain^23^. The decrements in neurotrophic factors such as BDNF and impaired hypothalamic–pituitary–adrenal axis (HPA axis) also are associated with depression^23^. In addition, immunologic activation including changes in inflammatory cytokines in hippocampus of brain, or peripheral blood and cerebrospinal fluid (CSF) is one of the persuasive hypothesis in pathophysiology of depression^24^. The kynurenine (KYN) pathway activation such as indoleamine 2,3-dioxygenase (IDO), kynurenine aminotransferase (KAT) and kynurenine monooxygenase (KMO, sometimes referred as kynurenine hydroxylase) link ‘cytokine theory’ and ‘monoamine hypothesis’ through tryptophan degradation and serotonin depletion^25,26^ and this pathway is associated with brain inflammation.

On the other hand, changes in ACE at the genetic level and the post-transcriptional level were reported to be associated with depression and glucocorticoid (Gc) secretion^27,28^. Moreover, an ANGII receptor blocker (ARB) showed anti-depressive effects^29^. However, despite previous researches, there is insufficient direct evidence of a relationship between ANGII and depression. Considered that chronically ANGII-infused mice showed increased blood pressure^30^, we implanted ANGII-containing osmotic pumps into male C57BL/6 mice subcutaneously to maintain elevated serum ANGII for 7 days (subacute) and 21 days (chronic). And then, behavioral evaluations and molecular analyses were conducted to elucidate the association between elevated serum ANGII and depression. Further, through these experiments, we aimed to explore the relationship of hypertension caused by elevated serum ANGII and depression.

## Results

### ANGII infusion over 21 days induced depressive-like behavior in mice

**Figure 1:**
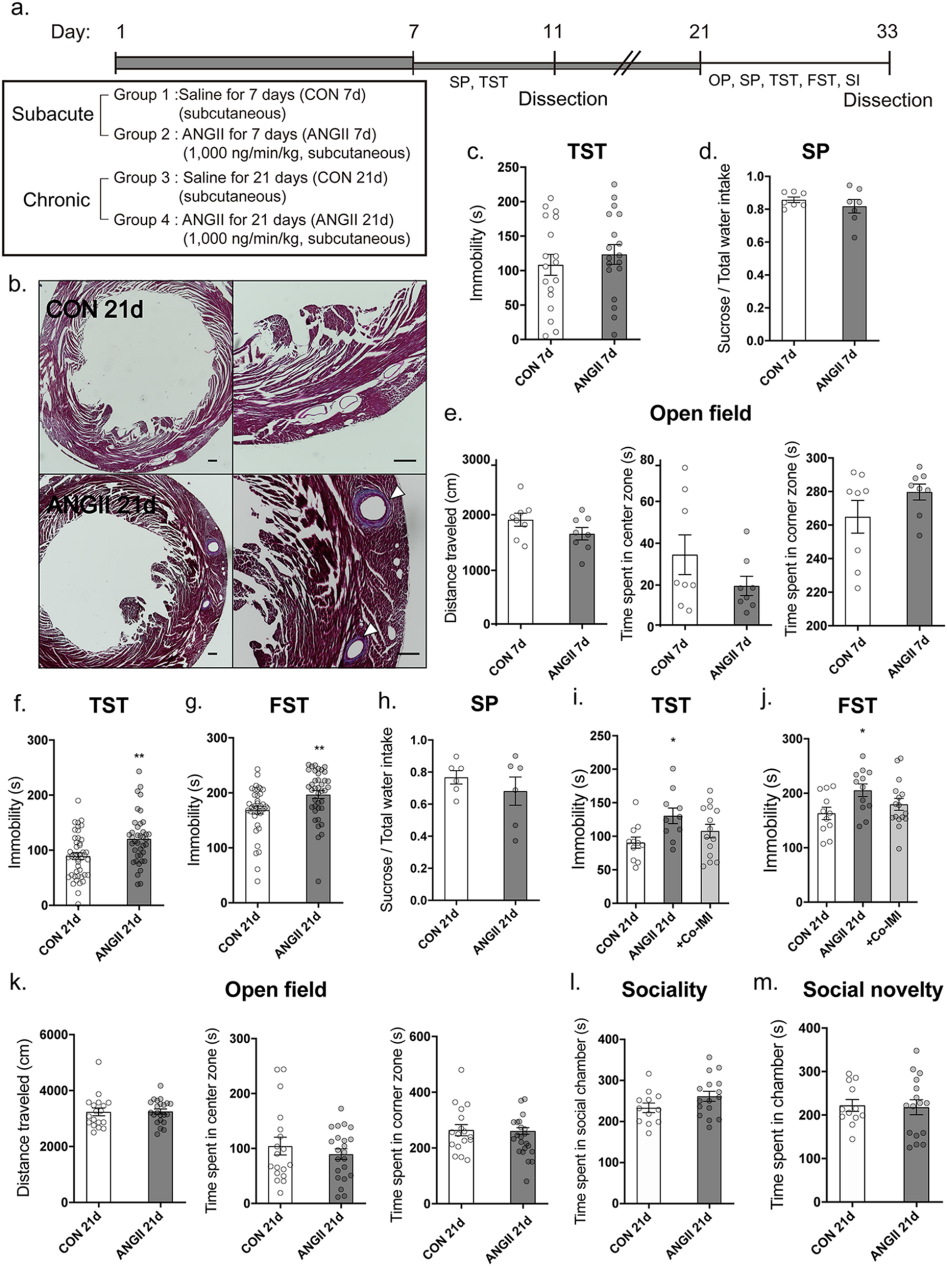
Chronically infused Angiotensin II (ANGII) induced depressive-like behaviors in mice. The mice were implanted with an osmotic pump containing saline or ANGII (1000 ng/min/kg) and a series of experiments was conducted following the time-course schedule (**a**). Representative heart images of control (CON 21d) and ANGII-infused mice (ANGII 21d) 21 days after implantation (**b**). Perivascular fibrosis/infiltration is marked with a white arrow. Scale bar = 200 μm. Mice were infused with ANGII for 7 days (subacute; 7d) and their behavior was assessed using the tail suspension test (TST), n = 18 in each group (**c**). Sucrose preference test (SP) for 1% sucrose solution over regular drinking water was examined for 2 days, after 2 days of habituation to two bottle conditions, and water intake/cage/day were checked (**d**), n = 7 (2–4 mice per cage) in each group. The open field test (**e**) was conducted to evaluate anxiety and results are presented as distance traveled, time spent in the center zone, and time spent in the corner zone, n = 8 in each group. Mice were infused with ANGII for 21 days (chronic; 21d) and their behavior was assessed using the TST (**f**), the forced swim test (FST) (g), n = 39–43 in each group, and SP (**h**), n = 6 (2–5 mice per cage) in each group. The effect of imipramine co-treatment (Co-IMI) during ANGII infusion for 21 days was assessed by TST (**i**) and FST (**j**), n = 10–16 in each group. The behavior of mice infused with ANGII for 21 days or more was assessed using the open field test (k), n = 17–22. The social behaviors of mice infused with ANGII for 21 days (ANGII 21d) were examined using the social interaction test and results are presented as sociality (l) and social novelty (m), n = 12–16. The data shown are mean ± SEM. *p < 0.05, **p < 0.01, and ***p < 0.001 compared to controls.

To evaluate the effect of ANGII, osmotic pumps containing ANGII (1000 ng/min/kg) were implanted into mice. Behavioral assessments were conducted after 7 days (7d; subacute) and 21 days (21d; chronic), and mice were sacrificed for histological and molecular analysis (Fig. 1a). The hearts of ANGII-infused mice were identified using Masson's Trichrome stain, which showed distinct changes including ventricular hypertrophy and perivascular infiltration / fibrosis 21 days after osmotic pump implantation (Fig. 1b). It seemed that 21 days of ANGII administration was sufficient to induce changes in the heart which are commonly seen in hypertensive state.

There were no significant changes in TST (Fig. 1c), SP (Fig. 1d) and open field test (Fig. 1e) between control (CON 7d) and ANGII-infused mice (ANGII 7d) at 7 days after ANGII capsule implantation (7d; subacute). However, at 21 days after ANGII capsule implantation (21d; chronic), ANGII-infused mice showed increased immobility time in the TST (*p* = 0.0013, CON 21d = 89.38 ± 5.929, ANGII 21d = 120.5 ± 7.372; Fig. 1f), and this depressive-like behavioral change was confirmed by the FST (*p* = 0.0060, CON 21d = 168.7 ± 6.973, ANGII 21d = 197.0 ± 7.194; Fig. 1g). However, there were no significant changes in SP between CON 21d and ANGII 21d mice (Fig. 1h).

There were significant differences among the three groups, CON 21d, ANGII 21d and + co-IMI in the ANOVA analysis and this showed that co-treatment of imipramine tended to decrease the increased immobility of TST (F = 3.495, *p* = 0.0424, CON 21d = 90.55 ± 8.316, ANGII 21d = 130.5 ± 11.65, + co-IMI = 107.7 ± 10.11; Fig. 1i) and FST (F = 3.145, *p* = 0.0551, CON 21d = 163.0 ± 11.46, ANGII 21d = 205.4 ± 11.62, + co-IMI = 179.6 ± 10.89; Fig. 1j) induced by ANGII. There were no significant changes in the open field test in these mice compared to controls, and it seemed that ANGII 21d mice did not exhibit any anxiety behaviors (Fig. 1k). Also, sociality and social novelty of ANGII 21d mice did not changed significantly in the social interaction test (Fig. 1l,m).

### Chronic ANGII infusion induced microglial activation in the hippocampus, and minocycline prevented ANGII-induced depressive-like behaviors

**Figure 2:**
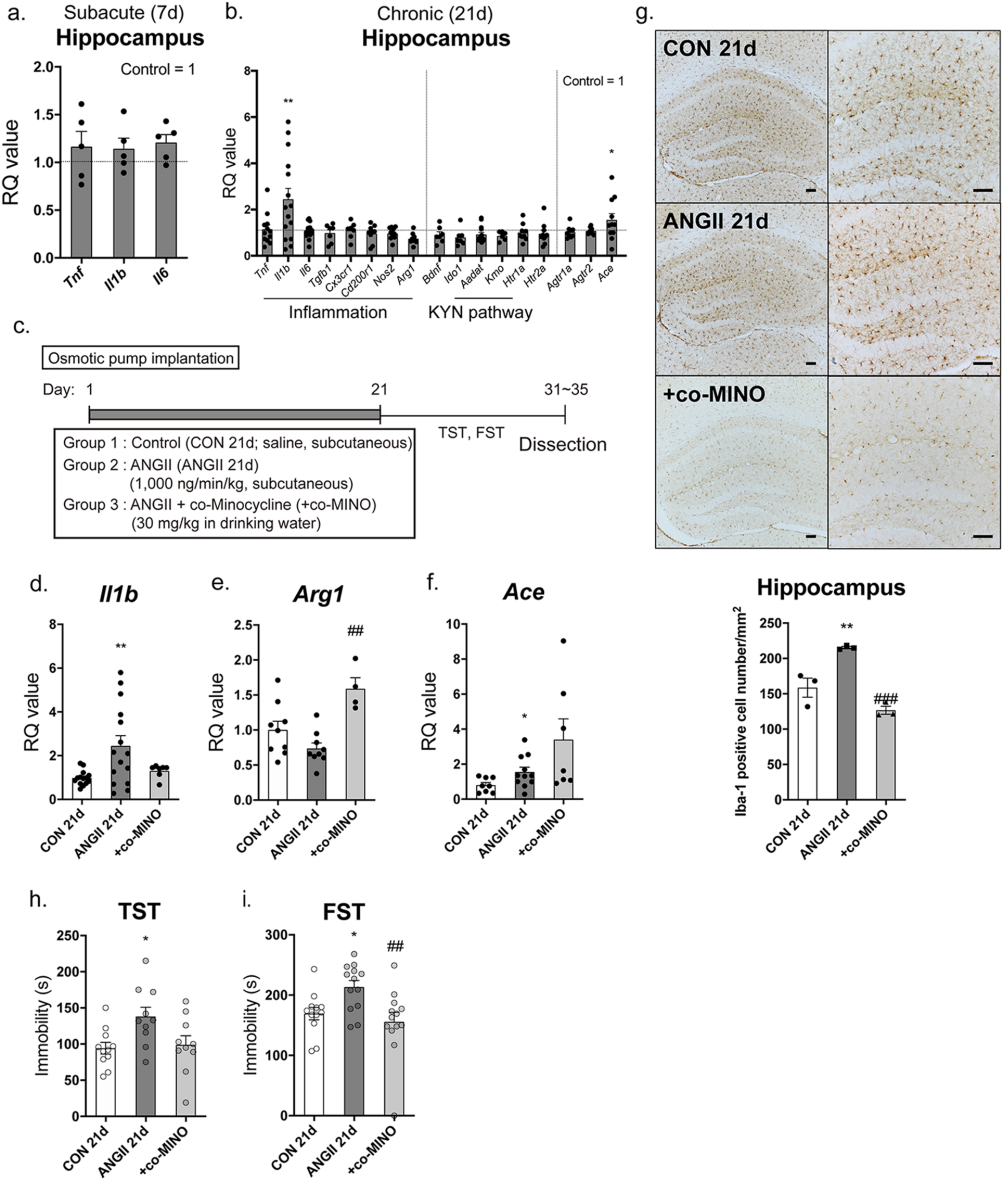
Chronically infused ANGII induced microglial activation in the hippocampus. The mRNA expression of immunologic markers such as *Tnf*, *Il1b,* and *Il6* in the hippocampus of the subacute ANGII-infused mice (7 days; ANGII 7d) was measured by qPCR (**a**). CT values were normalized to controls and RQ values are the ratio of respective transcription factors as a percentage of the controls, n = 5 in each group. Several markers of inflammation (*Tnf*, *Il1b*, *Il6*, *Tgfb1*, *Cx3cr1*, *Cd200r1*, *Nos2* and *Arg1*), a neurotrophic factor (*Bdnf*), kynurenine (KYN) pathway elements (*Ido1*, *Aadat* (KAT), *Kmo*), serotonin receptors (*Htr1a* and *Htr2a*) and RAAS elements (*Agtr1a*, *Agtr2* and *Ace*) were examined in the hippocampus of chronic ANGII-infused mice (21 days; ANGII 21d), n = 10–13 in each group (**b**). Minocycline was co-administered following the time schedule shown in (**c**) and the mRNA expressions of *Il1b* (**d**), *Arg1* (**e**), and *Ace* (**f**) were measured by qPCR. n = 4–15 in each group. An immunohistochemical study was performed to determine Iba-1 immunoreactivity in the hippocampus of each group of mice; 10 × (g, up, left) and 20 × (g, up, right) images were collected and the number of cells that are immunoreactive to Iba-1 were counted (**g**, down) in same regions of the hippocampus, n = 3 in each group. Scale bar = 100 μm. Behavioral assessments with the TST (**h**) and FST (**i**) were conducted in mice to confirm the effect of minocycline on behavior. n = 10–11 in each group. The data shown are mean ± SEM. *p < 0.05, **p < 0.01, ***p < 0.001, and ****p < 0.0001 compared to controls. ^#^*p* < 0.05, ^##^*p* < 0.01, and ^###^*p* < 0.001 compared to ANGII mice.

As ANGII can induce inflammation and inflammatory changes in the brain, especially in the hippocampus, which are associated with depressive behaviors, we hypothesized that ANGII-induced hippocampal inflammation could contribute to depressive-like behaviors. To confirm the inflammatory changes in the hippocampus, we measured the mRNA levels of inflammatory markers in the hippocampus first. There were no significant changes in the representative pro-inflammatory cytokines, *Tnf*, *Il1b,* and *Il6* in ANGII 7d mice (Fig. 2a). However, after 21 days from ANGII capsule implantation, ANGII 21d mice exhibited increased mRNA levels of *Il1b* (*p* = 0.0063, 2.448 ± 0.4704) and decreased pattern of mRNA levels of anti-inflammatory marker, *Arg1* (*p* = 0.0954, 0.7381 ± 0.0793; Fig. 2b). Other markers, known to be related to the pathophysiology of depression, such as *Bdnf* and kynurenine (KYN) pathway-related markers, were also measured but there were no significant changes between CON 21d and ANGII 21d mice. Similar to molecular analysis, histological analysis also showed an increased number of microglia (Iba-1 positive cells) in the hippocampus of ANGII 21d mice (*p* = 0.0080 in Tukey’s post hoc test, CON 21d = 158.6 ± 13.54, ANGII 21d = 215.3 ± 1.698; Fig. 2g).

The next question is whether depressive-like behaviors, which are associated with ANGII and hippocampal inflammation, have causal relationship. To solve this problem, minocycline, which inhibits microglial activation to suppress inflammation, was mixed into drinking water and co-administered during ANGII infusion (Fig. 2c). Minocycline effectively reversed the changes in *Il1b* (F = 5.666, *p* = 0.0077, ANGII 21d = 2.448 ± 0.4704, + co-MINO = 1.303 ± 0.1147; Fig. 2d) and *Arg1* (F = 10.11, *p* = 0.0010, ANGII 21d = 0.7361 ± 0.0793, + co-MINO = 1.589 ± 0.1577; Fig. 2e) mRNA and microglial activation (F = 27.59, *p* = 0.0009, + co-MINO = 126.7 ± 5.758; Fig. 2g) caused by ANGII infusion. In addition to molecular and histological changes, minocycline significantly reduced ANGII-induced depressive-like behavior in the FST (F = 5.815, *p* = 0.0066, CON 21d = 169.3 ± 10.37, ANGII 21d = 213.4 ± 10.75, + co-MINO = 155.6 ± 15.78; Fig. 2i) and also tended to decrease depressive-like behaviors in the TST (F = 4.512, *p* = 0.0200, CON 21d = 94.27 ± 8.108, ANGII 21d = 138.0 ± 12.89, + co-MINO = 98.90 ± 12.65; Fig. 2h).

The mRNA level of ACE was upregulated in ANGII mice despite ANGII infusion (*p* = 0.0444, 1.555 ± 0.2720; Fig. 2b). This increase was amplified when minocycline was co-administered (F = 4.456, *p* = 0.0231, + co-MINO = 3.403 ± 1.186; Fig. 2f).

### Chronic ANGII infusion did not affect peripheral cytokines level in mice

**Figure 3:**
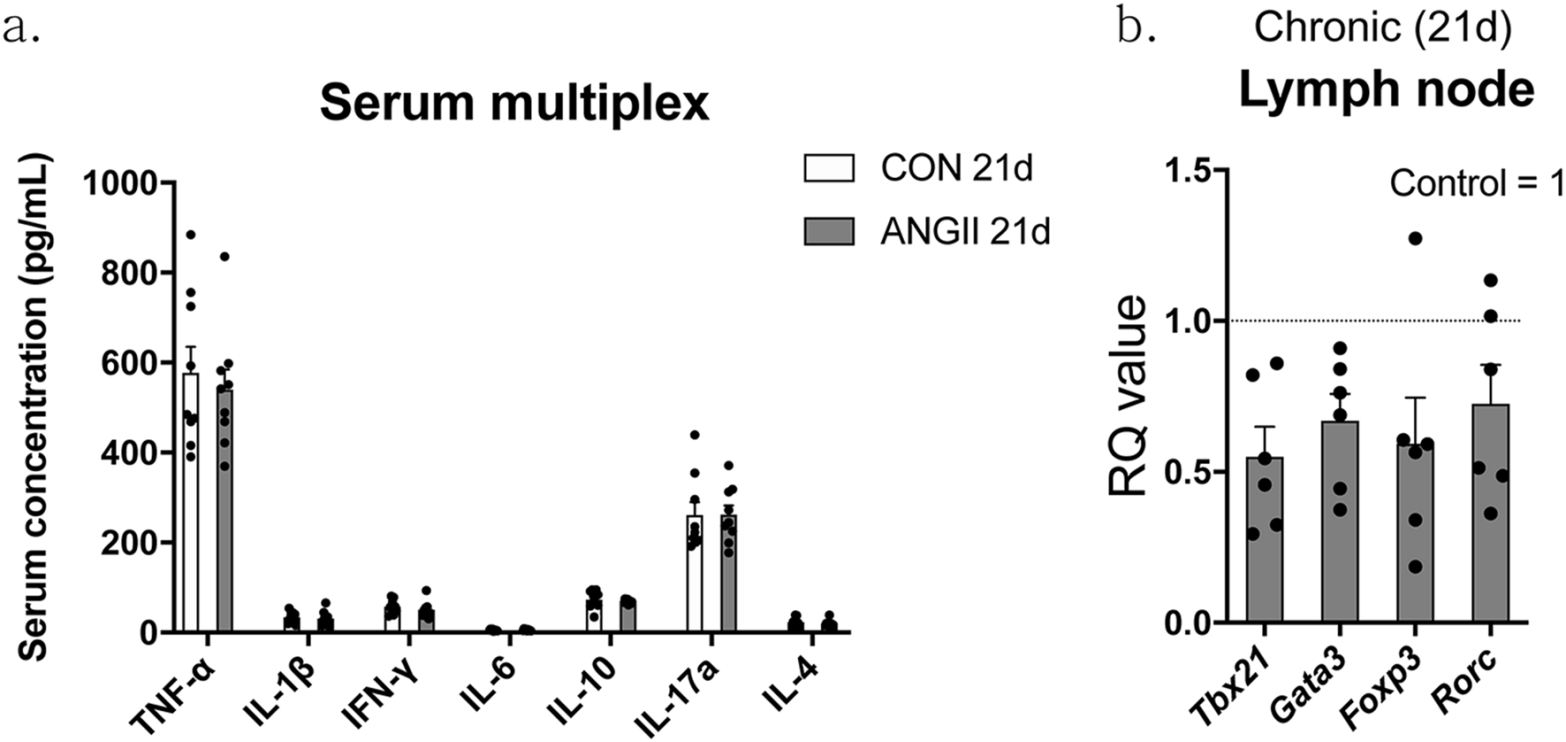
ANGII did not induce peripheral inflammatory changes in the mice. Serum from ANGII and control mice was obtained to measure pro- and anti-inflammatory cytokines, such as TNF-α, IL-1β, IFN-ɣ, IL-6, IL-10, IL-17a, and IL-4, using a Bio-Rad Bio-Plex assay, n = 9 in each group (**a**). Mesenteric lymph nodes were also dissected for qPCR to assess transcription factors of T Helper 1 cells (*Tbx21*), T Helper 2 cells (*Gata3*), regulatory T cells (*Foxp3*), and T Helper 17 cells (*Rorc*) (**b**). CT values were normalized to controls and RQ value are the ratio of respective transcription factors as a percentage of the controls, n = 6 in each group. The data shown are mean ± SEM. *p < 0.05 and **p < 0.01 compared to controls.

In addition to CNS inflammation, particularly in the hippocampus, increase in inflammatory markers in the peripheral system is one of the features observed in depressed patients^31^. To determine peripheral inflammation, we measured serum cytokines and mRNA levels of T cell markers in the mesenteric lymph node. No significant differences in serum cytokines were observed between CON 21d and ANGII 21d mice (Fig. 3a). On the other hand, lymph node T cell markers (e.g. *Tbx21*, *Gata3*, *Foxp3*, *Rorc*) tended to be all downregulated in ANGII mice (*Tbx21*, 0.5499 ± 0.09891; *Gata3*, 0.6700 ± 0.08817; *Foxp3*, 0.5936 ± 0.1521; *Rorc*, 0.7252 ± 0.1289; Fig. 3b).

### ANGII caused activation of the HPA axis and did not change the monoamine system

**Figure 4:**
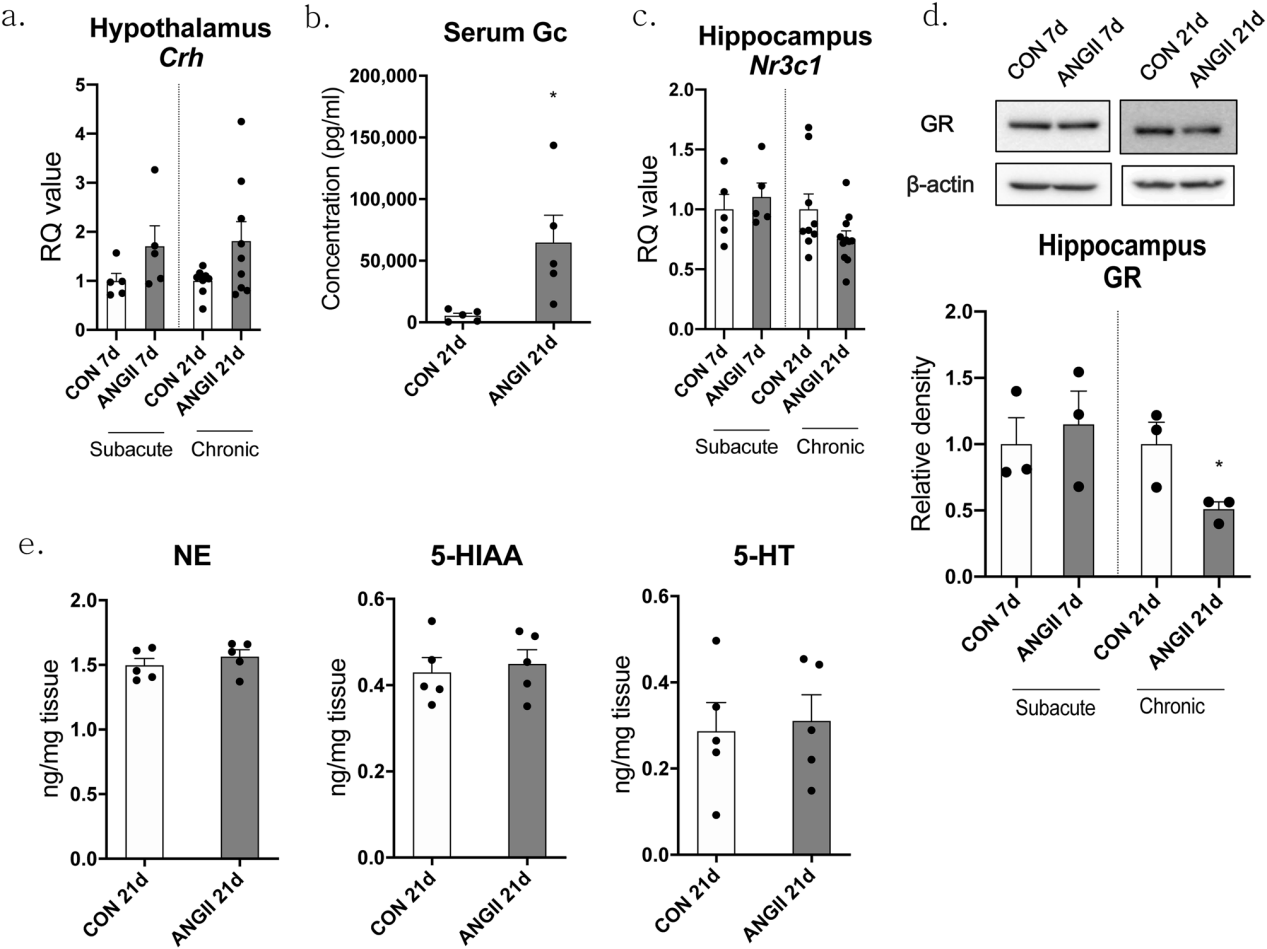
Chronically infused ANGII activated the HPA axis but had no effect on the monoamine system in the hippocampus. The expressions of *Crh* (corticotropin-releasing hormone) mRNA in the hypothalamus were assessed by qPCR (**a**) and the CT values were normalized to controls and RQ values are the ratio of respective transcription factors as a percentage of the controls, n = 5–10 in each group. Serum glucocorticoid (Gc) levels were measured by an EIA assay and n = 5 in each group (**b**). The expression of *Nr3c1* mRNA in the hippocampus was assessed by qPCR (**c**). The CT values were normalized to controls and RQ values are the ratio of respective transcription factors as a percentage of the controls, n = 5–11 in each group. GR protein levels of the hippocampus were assessed by western blot analysis and expression of GR was quantified using Image J (**d**). n = 3 in each group. The levels of norepinephrine (NE), 5-hydroxyindoleacetic acid (5-HIAA), and serotonin (5-hydroxytryptamine, 5-HT) were measured in hippocampus tissue of mice (e), n = 5 in each group. Data shown are mean ± SEM. *p < 0.05 and **p < 0.01 compared to controls.

In addition to inflammatory changes, HPA axis activity is another factor that needs to be investigated so that we can explain the depressive effect of ANGII. The components of the HPA axis such as hypothalamic corticotropin-releasing hormone (CRH) levels, serum Gc levels, and hippocampal glucocorticoid receptor (GR) levels were analyzed. The mRNA levels of hypothalamic *Crh* tended to be upregulated in both of ANGII 7d (*p* = 0.1515, 1.704 ± 0.4171), and ANGII 21d mice (*p* = 0.0632, 1.810 ± 0.3968; Fig. 4a). CRH stimulates pituitary gland to secrete ACTH, leading to the increase in serum Gc levels. So, we confirmed that the serum Gc level was actually increased compared to the control group by the increased CRH in ANGII 21d mice, as expected (*p* = 0.0284, CON 21d = 5390 ± 2055, ANGII 21d = 64,783 ± 22,152; Fig. 4b). The *Nr3c1* (GR) mRNA levels in the hippocampus did not show significant changes in ANGII 7d mice, but they tended to be downregulated in ANGII 21d mice (*p* = 0.0898, 0.7560 ± 0.064) in response to chronic Gc exposure (Fig. 4c). And this was confirmed at protein levels (21d, *p* = 0.0482, 0.5086 ± 0.05; Fig. 4d). Similar to KYN pathway-related factors (Fig. 2b), norepinephrine (NE), 5-Hydroxyindoleacetic acid (5-HIAA), and 5-hydroxytryptamine (5-HT) were not affected by ANGII in ANGII 21d mice (Fig. 4e).

### ARB reversed ANGII-induced depressive-like behaviors

**Figure 5:**
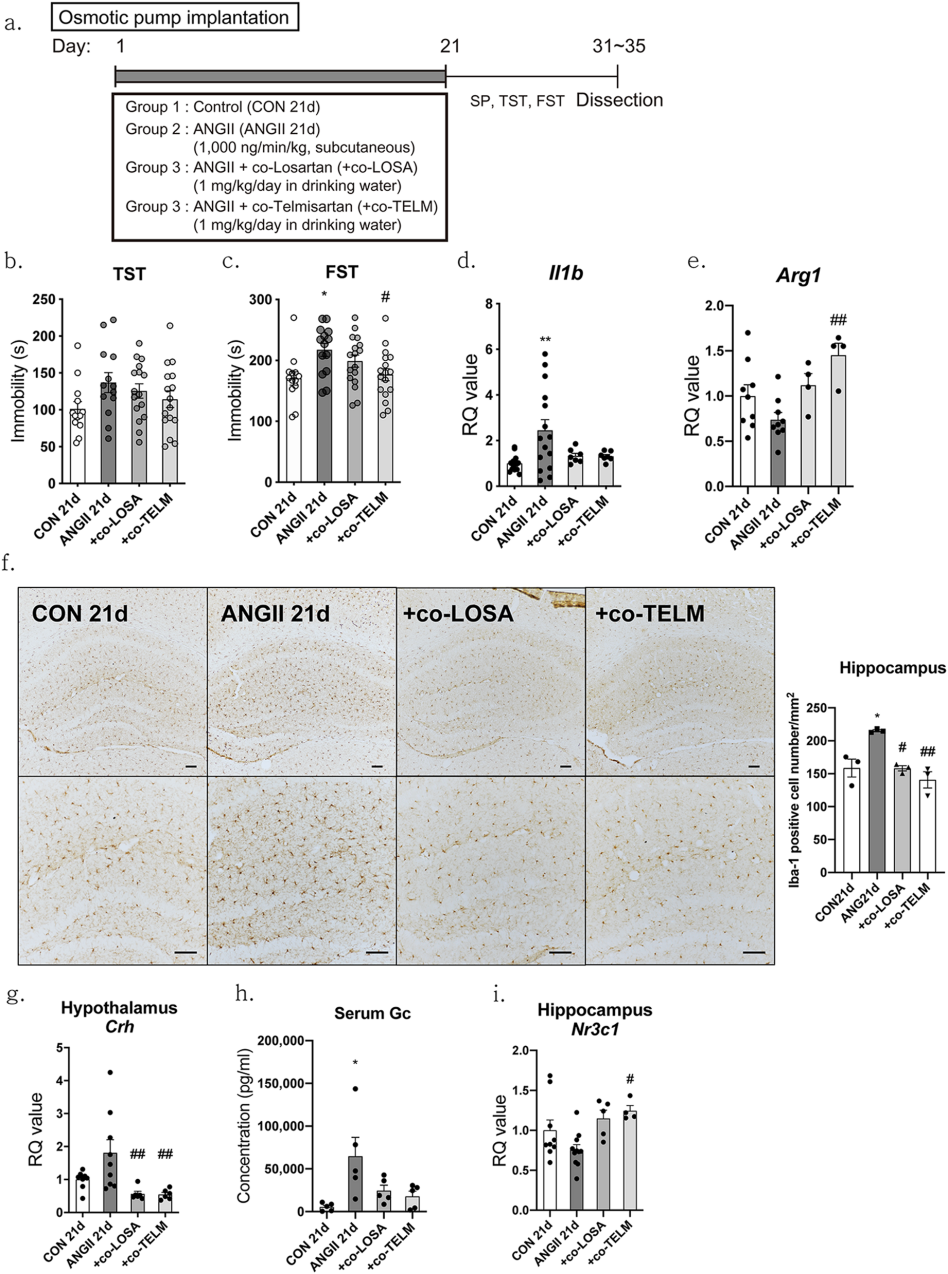
Telmisartan reversed ANGII-induced depressive-like behaviors and inflammation in the hippocampus. Losartan (1 mg/kg/day) and telmisartan (1 mg/kg/day) were co-administered in drinking water following the time schedule presented in (**a**) and behavioral assessments using the TST (**b**) and FST (**c**) were conducted. n = 11–17. Expression of *Il1b* (**d**) and *Arg1* (**e**) mRNA were measured by qPCR. CT values were normalized to controls and RQ values are the ratio of respective transcription factors as a percentage of the controls, n = 7–15 (**d**), 4–9 (**e**) in each group. An immunohistochemical study was performed to determine Iba-1 immunoreactivity in the hippocampus of mice, and representative images are shown at 10 × (f, left, up) and 20 × (f, left, down) magnification. Scale bar = 100 μm. The number of Iba-1 immunoreactive cells were counted in same regions of hippocampus (**f**, right), n = 3 in each group. The expression of *Crh* mRNA in the hypothalamus of mice was assessed by qPCR (**g**). CT values were normalized to controls and RQ values are the ratio of respective transcription factors as a percentage of the controls, n = 6–9 in each group. Serum Gc levels were measured by an EIA assay (**h**), n = 5 in each group. The expression of *Nr3c1* mRNA in the hippocampus was assessed by qPCR (**i**). CT values were normalized to controls and RQ values are the ratio of respective transcription factors as a percentage of the controls, n = 4–11 in each group. Data shown are mean ± SEM. *p < 0.05 and **p < 0.01 compared to controls. ^#^*p* < 0.05, ^##^*p* < 0.01, and ^####^*p* < 0.0001 compared to the ANGII 21d mice.

The next question is whether the series of changes induced by ANGII are really caused by ANGII directly or if another factor led to these changes. To answer this question, non-blood–brain barrier (BBB)-crossing ARB, losartan (+ co-LOSA; 1 mg/kg/day in drinking water) and BBB-crossing ARB, telmisartan (+ co-TELM, 1 mg/kg/day in drinking water), were co-administered with ANGII to determine if the drug could block the binding of ANGII and the ANGII receptor and inhibit the effect of ANGII (Fig. 5a).

Co-administration of losartan or telmisartan with ANGII, although not statistically significant, tended to reduce depressive-like behaviors induced by ANGII in TST (F = 1.558, *p* = 0.2105, CON 21d = 102.2 ± 10.68, ANGII 21d = 137.0 ± 13.47, + co-LOSA = 125.6 ± 9.798, + co-TELM = 114.3 ± 11.35; Fig. 5b) and FST (F = 4.001, *p* = 0.0118, CON 21d = 170.5 ± 11.04, ANGII 21d = 217.3 ± 10.69, + co-LOSA = 198.8 ± 9.988, + co-TELM = 176.5 ± 9.943; Fig. 5c). Also, changes of pro-inflammatory markers such as *Il1b* (F = 4.488, *p* = 0.0084, ANGII 21d = 2.447 ± 0.4708, + co-LOSA = 1.328 ± 0.1177, + co-TELM = 1.311 ± 0.0804; Fig. 5d) and *Arg1* (F = 5.491, *p* = 0.0057, ANGII 21d = 0.7381 ± 0.0793, + co-LOSA = 1.119 ± 0.1283, + co-TELM = 1.450 ± 0.1336; Fig. 5e) were reversed by losartan or telmisartan co-treatment. Additionally, increased microglial activity induced by ANGII were reversed by losartan or telmisartan co-treatment (F = 11.87, *p* = 0.0026, CON 21d = 158.6 ± 13.54, ANGII 21d = 215.3 ± 1.698, + co-LOSA = 158.1 ± 3.973, + co-TELM = 140.7 ± 12.44; Fig. 5f).

Furthermore, co-treatment with losartan or telmisartan attenuated HPA axis hyperactivation including hypothalamic *Crh* (F = 5.807, *p* = 0.0035, ANGII 21d = 1.810 ± 0.3968, + co-LOSA = 0.5652 ± 0.0774, + co-TELM = 0.5472 ± 0.0620; Fig. 5g), serum Gc (F = 6.760, *p* = 0.0048, CON 21d = 6623 ± 2123, ANGII 21d = 77,303 ± 23,593, + co-LOS = 24,522 ± 6396, + co-TELM = 17,857 ± 6106; Fig. 5h) and hippocampal GR (*Nr3c1*) (F = 4.248, *p* = 0.0148, ANGII 21d = 0.7560 ± 0.0644, + co-LOSA = 1.159 ± 0.1032, + co-TELM = 1.246 ± 0.065; Fig. 5i) induced by ANGII.

### ANGII induced inflammatory changes in microglial cell line

**Figure 6:**
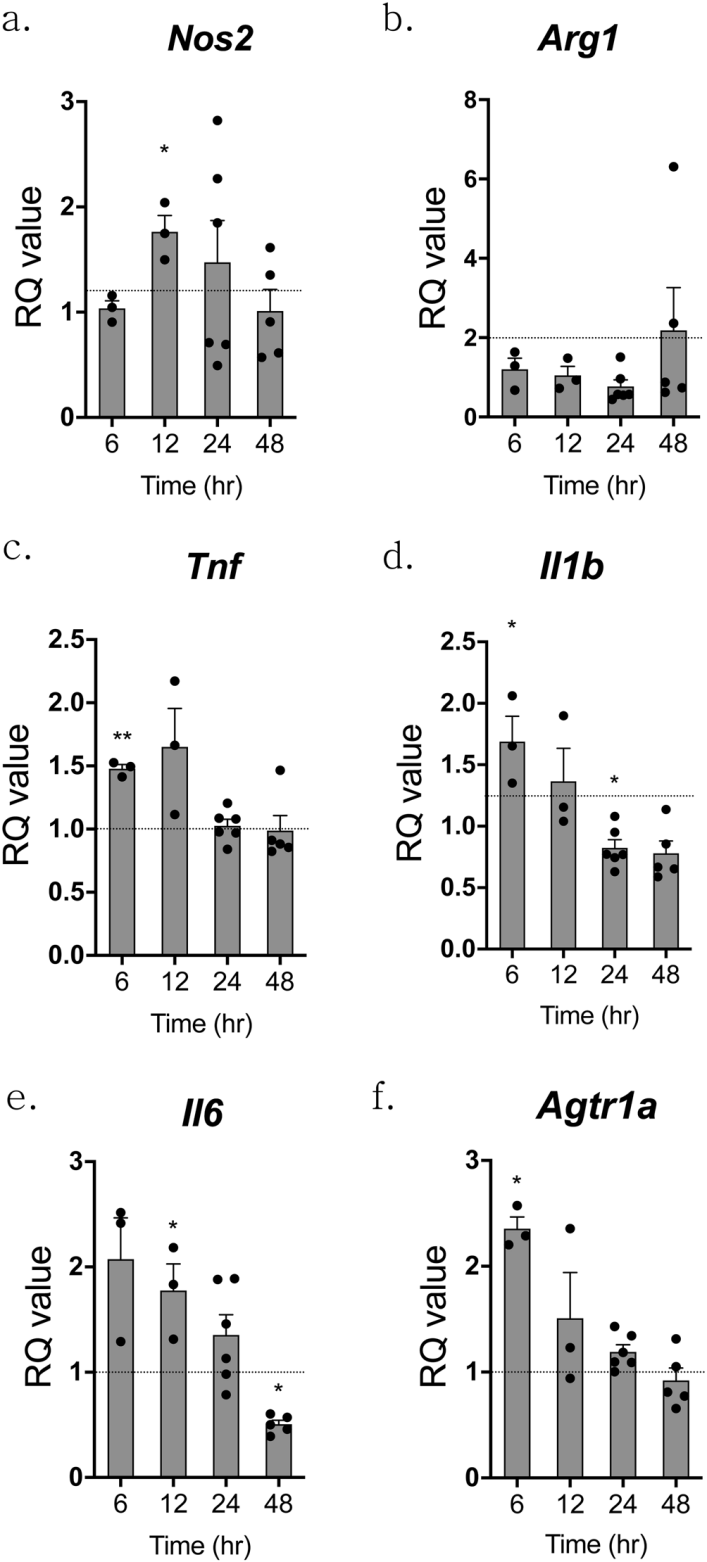
ANGII induced inflammatory changes in a microglial cell line. A murine microglial cell line, BV-2, was treated with 100 nM of ANGII for 6, 12, 24 and 48 h, and the mRNA markers associated with inflammation were assessed by qPCR and presented as change over time (**a**–**f**). The CT values were to controls and RQ values are the ratio of respective transcription factors as a percentage of the controls, n = 3–6 in each group. Data shown are mean ± SEM. *p < 0.05, **p < 0.01, ***p < 0.001, and ****p < 0.0001 compared to controls.

In addition to HPA axis hyperactivation, the direct effect of ANGII, which enter the brain through loosened BBB due to ANGII-induced hypertension, should be considered. Then, we identified the direct effect of ANGII on microglial cell by in vitro experiment using BV-2, a murine microglial cell line. Since ANGII had time-dependent effect in our in vivo experiment, the effects of ANGII (100 nM) on microglial cell were assessed at various time points (6, 12, 24 and 48 h). As shown in Fig. 6, ANGII induced increases of pro- or anti-inflammatory markers, such as *Nos2* (iNOS), *Arg1*, *Tnf*, *Il1b*, and *Il6*, at 6–12 h, and these effects were diminished over time. The mRNA levels of *Il1b* and *Il6* were still decreased at 48 h after ANGII treatment. Also, mRNA levels of *Agtr1a* (AT_1A_R), known to mediate the inflammatory effect of ANGII, were increased at 6 h after ANGII treatment and decreased over time. Consequently, it seemed that the inflammatory effects of ANGII on microglial cell line reached their peak between 6 and 12 h after ANGII treatment and gradually disappeared over time.

## Discussion

This is the first report demonstrating that ANGII can induce depressive-like behaviors which were specific to behavioral despair (TST and FST) via microglial activation in the hippocampus of mice. Some mechanisms appear to be associated with ANGII-induced depressive-like behavior. First, ANGII in periphery increases CRH expression in the hypothalamus PVN and consequently increases the stress hormone Gc in serum^32,33^. In normal condition, ANGII cannot cross intact BBB and ANGII exerts its effects through its receptors in the circumventricular organ (CVO), which lacks BBB, and consequently results in PVN activation^34^. PVN can also be stimulated via increase of sympathetic activity induced by ANGII^33^. Increased serum Gc due to CRH increase contributes to the microglial activation in the hippocampus and increased expression of *Il1b* as shown in other animal models for depression^23,35,36^.

Another possible mechanism is that ANGII directly increase pro-inflammatory cytokines, such as *Tnf*, *Il1b*, and *Il6*, in the microglial cells of the hippocampus, leading to depressive-like behaviors^37,38^ as seen out in vitro results. ANGII in the brain may have originated from peripherally infused ANGII which enter the brain parenchyma via impaired BBB which was damaged by sustained hypertension induced by itself^39,40^. Also, it is reported that systematically infused ANGII can cross BBB at a very low degree through the AT1R mediated transcytosis of BBB endothelial cells^41^. The newly synthesized ANGII in Brain RAS^42^ also can contribute to depressive-like behavior. Beyond these mechanisms, the secretion of inflammatory substances in the endothelium and astrocyte by ANGII may also be involved in microglia activation and depressive-like behaviors^43^, but there were no significant changes in astrocyte in our study, (supplementary Fig. f).

ANGII 21d mice (chronically ANGII-infused mice) exhibited inflammation in the hippocampus, including increased *Il1b* mRNA levels and microglial activation (Fig. 2b,g, supplementary Fig. h), while ANGII 7d mice (subacute) did not. It supports the theory that ANGII affects hippocampal microglial activation in a dose- and time-dependent manner^18^. The relationship between *Il1b* in the CNS and depression has been well-studied^44^, and the hippocampus in particular was thought to be closely linked to depression^45^. There are also evidences suggesting that the central administration of pro-inflammatory cytokines induced depressive behaviors^37,46^. Minocycline co-treatment with ANGII further clarified the association between hippocampal inflammation and ANGII-induced depressive-like behavior. Minocycline shows anti-inflammatory and neuroprotective effects through inhibition of microglial activation^47,48^. Similar to the findings of the preceding literature, co-treatment with minocycline in our study not only attenuated the depressive-like behaviors induced by ANGII infusion but also normalized alterations in *Il1b* and *Arg1* mRNA levels caused by ANGII infusion. Arginase (ARG-1), which metabolizes L-arginine to L-citrulline in innate immune cells, is known to be up-regulated in anti-inflammatory responses and contribute to tissue repair and proliferation^49^. Inducible nitric oxide (iNOS) and ARG-1 share a common substrate, L-arginine and are expressed mutually exclusive^50^. ANGII-induced inflammation was limited to the hippocampus and not exhibited in the hypothalamus and amygdala in our study (supplementary fig. g). Considering that the concept of region-specific microglia subtype has been suggested^51,52^, it is not surprising that ANGII induced inflammation limited to the hippocampus. Nevertheless, further research is needed on why inflammatory changes appear only in the hippocampus, not in the amygdala and hypothalamus.

Since BBB protect the brain from systemic circulation, it has been suggested that there will be a separated RAS (Renin-Angiotensin System) in the brain which is independent of peripheral system^53^. Peripherally infused ANGII can exert its effect through passage of loosened BBB or CVO which lacks BBB (peripheral pathway). In addition, locally synthesized ANGII in the brain RAS can in itself affect the brain (central pathway)^42^. To distinguish the mechanism of ANGII-induced hippocampal inflammation, two ARBs with different mechanisms of action were used in the experiment. Losartan and telmisartan are ARBs which have high binding affinity for AT1R than AT2R but losartan cross BBB poorly or not at all^54,55^, while telmisartan can cross BBB^56^. Co-treatment of losartan or telmisartan with ANGII blunted ANGII-induced depressive-like behaviors. Inflammatory changes induced by ANGII, including change of mRNA levels of *Il1b* and *Arg1* and microglial activation, were also normalized by both of ARBs co-administration. Both of two ARBs also normalized HPA axis activation caused by ANGII. Considering these, depressive-like behavior caused by ANGII-induced hippocampal inflammation seemed to be mediated by ANGII receptor signaling. In addition, considered that telmisartan can block both central and peripheral pathways, but losartan only can block peripheral pathway due to its poor BBB permeability, the action of ANGII seemed to occur via peripheral pathway.

Peripheral cytokines levels are associated with depression and stress vulnerability^31,57^ and are thought to be reliable biomarkers in depressed patients^31^. Increases in peripheral pro-inflammatory molecules can induce CNS inflammation by the humoral or neural pathways^53^. Since ANGII has pro-inflammatory properties^16,54^, we hypothesized ANGII would be involved in peripheral cytokines modulation. However, there was no change in the level of several key cytokines in ANGII 21d mice. Because a subset of T cells, such as regulatory T-cells (Treg), as well as cytokines are associated with depression^57^, we examined changes in T cell subtypes such as *Tbx21*, *Gata3*, *Foxp3*, and *Rorc* in mesenteric lymph node and found that these markers showed down-regulated pattern in chronically ANGII-infused mice. Contrary to several studies showing that ANGII treatment skewed T-cell subtypes to dominantly pro-inflammatory types^55,56^, our comprehensive results in vivo indicate that the effects of ANGII on the immune system can be affected by other mediators. And, in addition to direct effects, the immunological change seems to be affected by elevated serum Gc level. Gc has an immunosuppressive effect by suppressing T cell proliferation and cytokine secretion from T helper cells^58^. Thus, a combination of the pro-inflammatory effect of ANGII and anti-inflammatory effect of Gc could result in unchanged levels of serum cytokines in our study.

Based on our results, HPA axis hyperactivation seems to be another important property of chronic ANGII-infused mice. ANGII is known to not only increase sympathetic activity and stimulate to release ADH in the pituitary gland but also stimulate the hypothalamus directly to release CRH and vasopressin^32,33,59^. Aldosterone, a downstream hormone of ANGII, can also increase circulating Gc levels^60^. Studies showing that polymorphism of the *Ace* gene is associated with increased of serum Gc and depression supports our results^27,61^. Reduction of hippocampal *Nr3c1* mRNA also seems to be due to chronically increased serum Gc, which may lead to the GR insensitivity (Gc resistance) that is commonly exhibited in depressive states^62^. The hippocampus inhibit most aspects of HPA axis activity through GR so that hippocampal GR reduction can aggravate HPA axis hyperactivation^63^. This Gc resistance in the hippocampus seemed to be an important factor in depressive-like behaviors of ANGII 21d mice. Although hypothalamic *Crh* mRNA levels seemed to be already increased at 7 days after ANGII infusion and these trends of increase maintained until after 21 days from ANGII infusion, downregulation of hippocampal GR appeared not at 7 days but at 21 days. This is similar to the behavioral test pattern that depressive-like behavior did not appear at subacute phase, but it appeared at chronic phase. Additionally, reduced *Nr3c1* level in hippocampus is common feature with chronic stress models^64^.

HPA axis hyperactivation and hippocampal microglial activation are not independent phenomenon in ANGII 21d mice. Gc is known to show anti-inflammatory effect and limit microglial activation during acute stress^65^. However, when administered with high level or chronically, Gc is reported to enhance microglial activation instead and to prime the microglia to immunologic stimulation^66,67^. Chronically elevated Gc decreased hippocampal GR and absence of GR in microglia resulted in activated microglia which can cause neuronal death^68^. Considering this, chronically elevated Gc level caused by HPA axis hyperactivation contributed to microglial activation in the hippocampus. In addition, inflammatory cytokines secreted by activated microglia can reduce hippocampal GR level^69^.

Our results also showed that co-treatment of minocycline inhibited HPA axis activation in ANGII 21d mice (supplementary Fig. c–e). Minocycline is reported to exhibit neuroprotective effect via suppressing microglial activation and glutamatergic neurotransmission^70,71^. It is thought that minocycline protected the hippocampus from inflammatory damage and consequently, intact hippocampal GR contributed to prevention of HPA axis hyperactivation. Further, given that minocycline can suppress glutamatergic neurotransmission, it is speculated that minocycline inhibited glutamatergic activation of hypothalamic PVN neuron and resulted in decrease of CRH release. Because of this effect, minocycline seems to have normalized the HPA axis despite the ANGII infusion.

On the other hand, monoamines such as NE, HIAA, and 5-HT were not altered in the hippocampus of ANGII 21d mice, although the monoamine theory is the well-established hypothesis from early clinical observation in depression pathophysiology^23^. Despite microglial activation, the mRNA level of KYN pathway-related genes were also not changed. These results are similar to those of our previous studies^26,57^. Taken together, chronic elevation of ANGII is suggested to be a risk factor for specific forms of depression.

The possibility that ANGII will directly enter the brain parenchyma and have an effect should not be ruled out. Increased BBB permeability by sustained hypertension allows more ANGII to enter the brain than under normal condition. Similar to previous studies showing that ANGII has a pro-inflammatory effect in microglia^72^*, *in vitro administration of ANGII increased pro-inflammatory cytokines in a microglial cell line, but these changes were not persistent. *Il1b* and *Il6* mRNA level were reduced over time, indicating that microglia have a self-regulatory system against changes in the microenvironment (e.g., cytokine elevation). However, this self-regulatory system seems to not function well in chronically ANGII-infused state. This discrepancy can be explained by the fact that, in contrast to an in vitro cell line, microglia in vivo interact with various types of cells, such as astrocytes, neurons, and oligodendrocytes. Since astrocytes have abundant ANGII receptors, they might be sensitive to ANGII^73,74^, but astrocyte was not activated in our result. Additionally, systemically-infused ANGII stimulates inflammatory mediator release, such as superoxide^75^, in the endothelial cells that composed the BBB, and this inflammatory mediator can influence microglia indirectly.

Considering these, we concluded that the molecular and behavioral changes induced by ANGII resulted from HPA axis hyperactivation through CVO and ANGII penetration through loosened BBB from the plasma. It is not clear that which of these two pathways play the main role in depressive-like behaviors. However, considering that the direct effect of ANGII on microglial cells has decreased over time in vitro study, the effect through CVO might have more contribution rather than direct effect on microglia. However, further in vivo study seems to be needed.

There are some limitations in our study. We did not confirm directly whether ANGII infusion did increase blood pressure in mice. However, despite that there is no evidence of direct measurement, ANGII-induced hypertension is well-established hypertension model in animal. The study using same experimental model (subcutaneous ANGII infusion at 1000 ng/min/kg by osmotic pump) showed increase of blood pressure within hours of pump implantation and these increases were maintained until 28 days after pump implantation^30^ . In addition, we checked ANGII-induced changes in the heart to overcome shortcoming and found that ANGII 21d mice exhibit hypertensive structural changes in the heart such as ventricular hypertrophy and perivascular fibrosis. Although ANGII alone can cause hypertrophy and perivascular fibrosis independent of blood pressure^76^, structural changes in the heart is a still significant factor in inferring physical changes associated with ANGII-induced hypertension.

Another limitation is that the minocycline used in our experiment may have other effects than inhibition of microglial activation. Minocycline is an antibiotic and is reported to alter behavior, microglial activity and gut microbiota^77^. With the concept of ‘gut-brain axis’ being proposed, the possible relationship between gut microbiome dysfunction and depression is drawing attention^78^. Given this, careful approach is likely to be needed in interpreting the experimental results caused by minocycline. Also, telmisartan which was used as BBB-crossing ARB in our study is reported to have partial agonistic effect on Peroxisome Proliferator-Activated Receptor (PPAR)γ^79^. Given that PPARγ activation suppresses inflammation^80^ and may have antidepressant effect^81^, the results of the Telmisartan co-treated mice might be the result of its involvement in the AT1R blocker function of Telmisartan as well as PPARγ activation, so care should be taken to interpret it.

**Figure 7:**
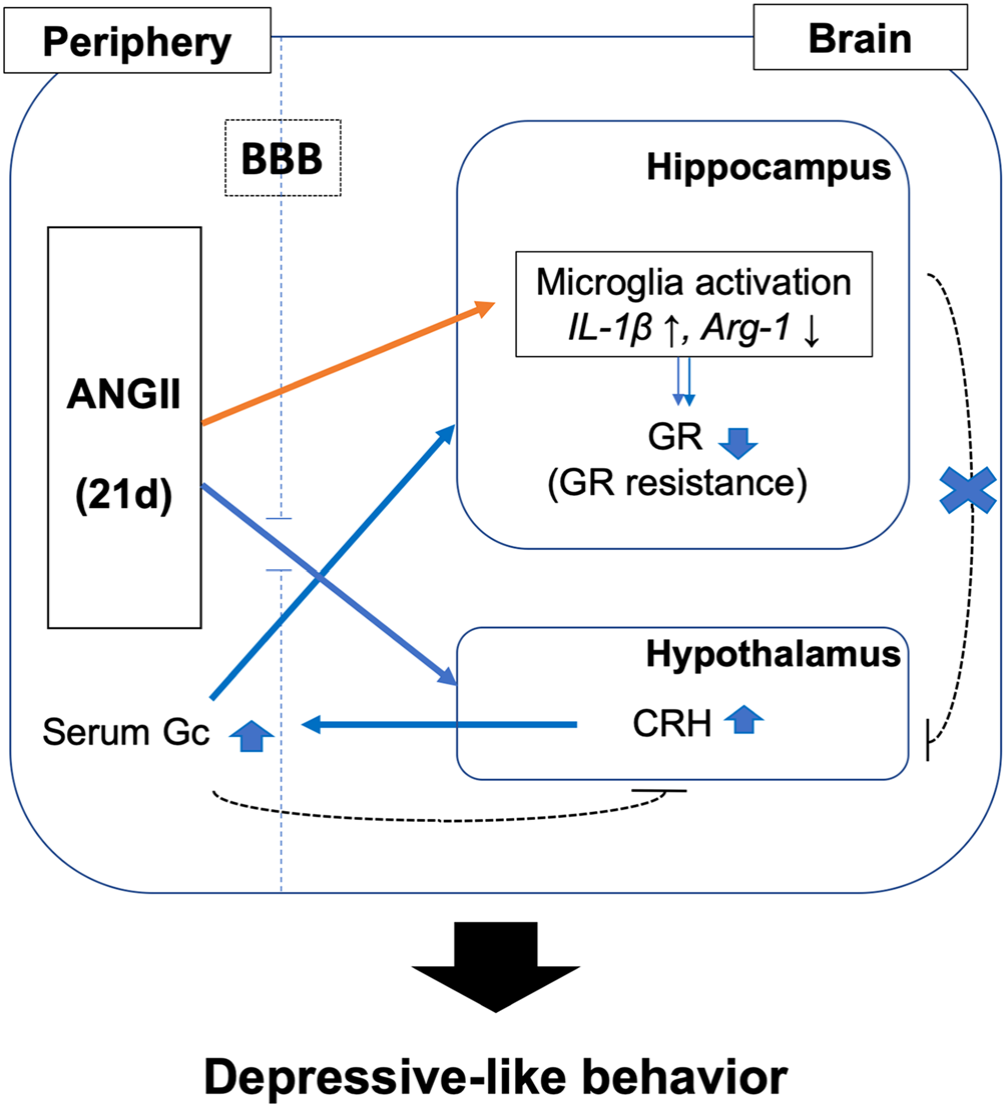
Schematic representation of ANGII-induced depressive-like behaviors. Chronic infused ANGII increases CRH expression in the hypothalamus and results in increase of stress hormone Gc by activating the HPA axis. ANGII also can directly activate microglial cells in the hippocampus which can contribute to HPA hyperactivation. Collectively, ANGII can lead to depressive-like behaviors in mice.

In summary, chronic ANGII can induce depressive-like behaviors via hippocampal inflammation and HPA axis activation (Fig. 7). Our results suggest that chronic elevation of ANGII could be a risk factor in depression, via hippocampal inflammation. Despite several reports stating that ANGII induces brain inflammation, this is the first study to report that ANGII-induced inflammation in the hippocampus can lead to depressive-like behaviors in mice. ANGII can be elevated by RAAS system activation as a compensation mechanism against decreases in renal perfusion, which is associated with hypertension. Thus, control and management of blood pressure in hypertensive patients seems to be important for prevention of comorbid depression.

## Material and methods

### Experimental animals

All animal care and procedures complied with the Animal Welfare Act and were in accordance with institutional guidelines and approved by the Institutional Animal Care and Use Committee (IACUC) of the CHA University and have been performed in accordance with the relevant guidelines and protocol (IACUC180173). Male C57BL/6 mice (Orient Bio Inc. Seoul, Korea), age 7 weeks weighing 20–25 g were used for all experiments. Animals were housed five per cage under SPF conditions maintained at 22 ± 0.5 °C with an alternating 12-h light–dark cycle at CHA BIO COMPLEX animal facility. Food and water were available ad libitum. Animals were allowed to acclimate to the laboratory for a week before the beginning of the experiments. To reduce variation, all experiments were performed during the light phase of the cycle.

### Osmotic minipump implantation and drug treatment

Mice were randomly assigned to control or experimental groups in cage units. ANGII was administered to mice by osmotic minipump implantation to maintain consistent levels of serum ANGII. Alzet osmotic minipumps (model 2006, Durect Corporation, Cupertino, USA) were subcutaneously implanted as previously described^18^. Animals were anesthetized via inhalation of isoflurane before minipump implantation and observed until fully recovered. Each pump delivered 1000 ng/min/kg of ANGII (Sigma, St. Louis, MO, USA) at a rate of 0.15 μl/h, following the experimental schedules outlined in Fig. 1a, 2c and 5a. Saline (0.9%)-containing osmotic minipumps were subcutaneously implanted for the control mice. In addition, imipramine, minocycline, losartan and telmisartan were administrated with drinking water to avoid stress due to drug injection.

#### Experiment 1

To determine time-dependent effects of ANGII administration, behavioral measurements were conducted in mice 7 days and 21 days after pump implantation (Fig. 1a). To evaluate the effect of antidepressants on ANGII-induced depressive-like behaviors, imipramine (Sigma-Aldrich, St. Louis, Missouri; 20 mg/kg/day) was dissolved in drinking water and administered during the behavioral assessment period to avoid stress due to drug injection.

#### Experiment 2

To evaluate the effects of microglial inhibition on ANGII-induced changes, minocycline (TCI, Tokyo, Japan; 30 mg/kg/day) was dissolved in drinking water and administered to mice for 21 days or more to avoid stress due to drug injection. Minocycline treatment was continued during the behavior assessment period to avoid stress due to drug injection (Fig. 2c).

#### Experiment 3

Losartan (Sigma-Aldrich, St. Louis, Missouri; 1 mg/kg/day) and telmisartan (Sigma-Aldrich, St. Louis, Missouri; 1 mg/kg/day) was dissolved in drinking water to administer to mice for 21 days, or more, after pump implantation to avoid stress due to drug injection. Losartan and telmisartan treatment were continued during the behavior assessment period (Fig. 5a).

### Behavioral testing

Behavior testing was performed according to our previous studies^26^. Mice were allowed to acclimate to a testing room for at least 30 min before the experiments. All experiments were conducted during the light cycle, between 9:00 AM and 5:00 PM, in a series, with one experiment per day. The open field test and social interaction test were performed using EthoVision XT9 video tracking system (EthoVision Version 9, Noldus, Netherlands).

- *Tail suspension test (TST)* The TST was performed as previously described to measure spontaneous activity in rodents. The apparatus consisted of a cupboard with a hook attached to the top. Mice were suspended by securing the tail to the hook by wrapping adhesive tape around the tail. This was done carefully to ensure not to fold the tail, and the tip of the tail was wrapped 2 cm away from the top of the hook. The data of the mice that climbed their tails were removed from the test. Mice were hung for 7 min, and the immobility time was measured. Observers were blinded to the groups and the immobility time was measured and compared by two observers to minimize bias.
- *Forced swimming test (FST)* In the FST, we assessed the ability of mice to cope with an inescapable, stressful situation, which is affected by depressive-like behavior. FST was performed to measure spontaneous activity in rodents. Mice were individually placed in a 2 L Pyrex beaker (13 cm diameter, 24 cm height), filled with 23 °C water, with a depth of 17 cm. All mice were forced to swim for 6 min, and the duration of immobility was measured during the final 5 min of the test. Immobility was defined as the time that the mouse spent floating without struggling and making only the movements necessary to keep its head above the water level. Observers were blinded to the groups and the immobility time was measured and compared by two observers to minimize bias.
- *Sucrose preference test (SP)* Preference for sucrose solution over drinking water was measured to assess anhedonia. Preference for sucrose is usually decreased in depressed mice. To assess SP, mice were provided with two bottles filled with 1% sucrose, diluted in drinking water or drinking water alone. Animals were acclimatized to two bottle conditions for two consecutive days and were their preference was assessed over two additional days. The position of the bottles was interchanged during the 4 days of testing. On each test day, the fluid levels were noted per cage. SP was calculated as percentage of sucrose/total fluid consumed.
- *Open field test* The open field test was performed to measure spontaneous activity in rodents. The apparatus consisted of a white Plexiglas box square, 50 cm × 50 cm × 40 cm, divided into nine equal squares. Each mouse was placed in the center of the floor, and, after 30 s of adaptation, the total distance traveled and the time spent in center zone and four corner zones was recorded and measured using EthoVision XT9 video tracking system over 10 min. After each test, the arena was cleaned with 70% alcohol solution.
- *Social interaction test (SI)* The social interaction box is a rectangular, three-chambered box made of white Plexiglas (60 cm × 40 cm × 40 cm). Each chamber is 20 cm × 40 cm × 40 cm. Dividing walls were made from clear Plexiglas, with a small opening (square, 6 cm × 6 cm), allowing access into each chamber. The test mouse was first placed in the middle chamber and allowed to explore for 5 min. The doorways into the two side chambers were blocked with plastic boxes during this habituation phase. After 5 min of habituation, an unfamiliar mouse, which was of same strain and had no prior contact with the subject mouse, was placed in one of the side chambers. The location of the stranger mouse in the left vs. right side chamber was systematically alternated between trials. The stranger mouse was enclosed in a cylinder made of transparent Plexiglas, which allowed nose contact through the holes, but prevented fighting. The cylinder was 15 cm in height with a bottom diameter of 8 cm and holes spaced 1 cm apart. The cover, made of Plexiglas, was placed on the top of the cylinder to prevent climbing by the test mice or escape of the stranger mouse. Both doors to the side chambers were then unblocked and the test mouse was allowed to explore the entire social interaction box for a 10-min session to measure sociality. Activity in the social interaction chamber was recorded, and time spent in each chamber was measured using EthoVision XT9 video tracking system. At the end of the first 10 min, each mouse was tested in a second 10-min session to check social preference for a new stranger. A second unfamiliar mouse, also enclosed in a cylinder made of transparent Plexiglas, was placed in the chamber that had been empty during the first 10-min session. The test mouse had a choice between the first, already-investigated, unfamiliar mouse (stranger 1), and the novel, unfamiliar mouse (stranger 2). As described above, activity in the social interaction chamber was recorded, and time spent in each chamber was measured using EthoVision XT9 video tracking system.

### BV-2 microglial cell line cultures and ANGII treatment

Murine BV-2 microglial cells were maintained in Dulbecco’s modified Eagle medium (DMEM) supplemented with 10% fetal bovine serum and antibiotics at 37 ^◦^ C in a humidified incubator under 5% CO_2_. Then cells were seeded into 6-well plates with 2 × 10^5^ cells/well for qPCR analysis. After the serum starvation for overnight, the cells were treated with media (control) or 100 nM of ANGII^72^ for 6, 12, 24 or 48 h.

### Molecular works

At least 3 days after the last behavioral test, animals were sacrificed by terminal anesthesia (pentobarbital; IP, 100 mg/kg) within 5 min of entering the room between 14:00 and 16:00. Whole blood was collected by cardiac puncture and the serum was isolated and stored at − 80 °C until assayed. The tissues were also dissected and stored at − 80 °C until assayed. For perfusion, all mice were first deeply anesthetized with pentobarbital (100 mg/ kg, i.p.), and perfused through the heart with physiological saline followed with ice-cold phosphate-buffered 4% paraformaldehyde (pH 7.4).

Molecular work was performed according to our previous studies^26^. (a) Quantitative reverse transcriptase polymerase chain reaction (qRT-PCR), (b) Total protein extraction and Western blot analysis, (c) Immunohistochemistry (d) Determination of serum chemistries, (e) Quantification of serotonin (5-hydroxytryptamine, 5-HT) and its metabolite, and (f) 5-hydroxyindoleacetic acid (5-HIAA) by high pressure liquid chromatography and electrochemical detection (HPLC-ECD) were described in supplementary material and methods. Primer information for qRT-PCR was shown in Table 1.

**Table 1:** Primer information.

Primers	Forward (5′ → 3′)	Reverse (5′ → 3′)
*Tnf*	GAGTCCGGGCAGGTCTACTTT	CAGGTCACTGTCCCAGCATCT
*Il1b*	GGCTGGACTGTTTCTAATGC	ATGGTTTCTTGTGACCCTGA
*Il6*	CCACTTCACAAGTCGGAGGCTTA	GCAAGTGCATCATCGTTGTTCATAC
*Tgfb1*	TGACGTCACTGGAGTTGTACGG	GGTTCATGTCATGGATGGTGC
*Cx3cr1*	TGGCCCAGCAAGCATAG	CATGTCTGCTACCCTCACAAA
*Cd200r1*	AAATGCAAATTGCCAAAATTAGA	GTATAGCTAGCATAAGGCTGCATTT
*Nos2*	CATTGGAAGTGAAGCGTTTCG	CAGCTGGGCTGTACAAACCTT
*Arg1*	CTCCAAGCCAAAGTCCTTAGAG	AGGAGCTGTCATTAGGGACATC
*Bdnf*	TGCAGGGGCATAGACAAAAGG	CTTATGAATCGCCAGCCAATTCTC
*Ido1*	TGTGAATGGTCTGGTCTC	CTGTGCCCTGATAGAAGT
*Aadat*	CCAGGAACCCTTTATGCTATGAA	TGGAATAATCCCATGCTCATCA
*Kmo*	GGTCGCCTTCACCAGAATAA	ATCCAGGCAGGTCTTCTCAA
*Htr1a*	CTGTTTATCGCCCTGGATG	ATGAGCCAAGTGAGCGAGAT
*Htr2a*	CCGCTTCAACTCCAGAACCAAAGC	CTTCGAATCATCCTGTACCCGAA
*Agtr1a*	GGACACTGCCATGCCCATAAC	TGAGTGCGACTTGGCCTTTG
*Agtr2*	GCACCAATGAGTCCGC	AGGGAGGGTAGCCAAA
*Ace*	GGATACCTACCCTTCCTACATCAGC	CTACCCCACATATCACCAAGCA
*Tbx21*	CAACAACCCCTTTGCCAAAG	TCCCCCAAGCAGTTGACAGT
*Gata3*	AGAACCGGCCCCTTATCAA	AGTTCGCGCAGGATGTCC
*Foxp3*	GAACCCAATGCCCAACCCTAG	TTCTTGGTTTTGAGGTCAAGGG
*Rorc*	ACTACGGGGTTATCACCTGTGAG	GTGCAGGAGTAGGCCACATTAC
*Crh*	GTTAGCTCAGCAAGCTCACAG	GCCAAGCGCAACATTTCATTT
*Nr3c1*	GGATGCCATTATGGGGTCCT	TCGTTTTTCGAGCTTCCAGGT

### Statistical analysis

The data were presented as the mean ± standard mean error (SEM). The statistical significance of differences between groups was assessed with Student’s t test and one-way analysis of variance (ANOVA) using GraphPad Prism version 7 for Mac (GraphPad, La Jolla, CA). Tukey’s post hoc test was performed when *p* values were < 0.05. *p* < 0.05 was considered as statistically significant.

### Ethics approval and consent to participate

All experimental procedures were approved by the Institutional Animal Care and Use Committee (IACUC) of the CHA University (IACUC180173).

## Supplementary Information

Supplementary Information.

## Data Availability

All data generated and/or analyzed during the current study are available from the corresponding author on reasonable request.

## Abbreviations

Angiotensin II: ANGII
RAAS: Renin–angiotensin–aldosterone system
ACE: Angiotensin converting enzyme
AT_1_R: Angiotensin II receptor type 1
AT_2_R: Angiotensin II receptor type 2
Gc: Glucocorticoid
HPA axis: Hypothalamic–pituitary–adrenal axis
ARB: Angiotensin II receptor blocker
TST: Tail suspension test
FST: Forced swimming test
SP: Sucrose preference test
SI: Social interaction test
TNF-α: Tumor necrosis factor alpha
IL-1β: Interleukin-1 beta
IL-6: Interleukin 6
BDNF: Brain-derived neurotrophic factor
KYN: Kynurenine
Iba-1: Ionized calcium-binding adapter molecule 1
ARG-1: Arginase 1
CNS: Central nervous system
Foxp3: Forkhead box P3
Rorc: RAR-related orphan receptor C
CRH: Corticotrophin-releasing hormone
GR: Glucocorticoid receptor
NE: Norepinephrine
5-HIAA: 5-Hydroxyindoleacetic acid
5-HT: 5-Hydroxytryptamine
BBB: Blood–brain barrier
TELM: Telmisartan
Treg: Regulatory T-cell
