# Supplementary material for: Chronically infused angiotensin II induces depressive-like behavior via microglia activation
Source: Sci Rep. 2020 Dec 16;10:22082. doi: 10.1038/s41598-020-79096-2 (PMC7744531; doi:10.1038/s41598-020-79096-2)
Supplement: Supplementary file 1 — Supplementary Information. [file 41598_2020_79096_MOESM1_ESM.pdf]

Supplementary Material

To

Chronically infused angiotensin II induces depressive-like behavior via microglia activation

Hyun-Sun Park, Min-Jung You, Bohyun Yang, Kyu Beom Jang, Jongman Yoo, Hyun Jin Choi, Sang-Hyuk Lee, Minji Bang, Min-Soo Kwon

Supplementary Materal and methods.

a) Quantitative reverse transcriptase polymerase chain reaction (qRT-PCR).

The hippocampus, hypothalamus and the mesenteric lymph nodes were dissected after ANGII infusion by implantation of osmotic pump for 21 days followed by series of behavioral test. Also, BV-2 microglial cells treated with media or ANGII were washed with ice cold PBS. The frozen tissue was homogenized in 1 mL of Trizol reagent per 100 mg of tissue (Invitrogen, TX, USA) and the cells were added with 1 mL of Trizol per well for RNA extraction. Chloroform was added to separate the phase that contains RNA, and isopropyl alcohol was added to precipitate RNA. The precipitated RNA pellet was re- dissolved in DEPC- treated water (Bioneer, Seongnam, Korea) after air-drying the pellet. Quantification of RNA concentration was determined by the absorption at 260 nm. One microgram of messenger RNA (mRNA) was reverse-transcribed into cDNA in 20 μL of reaction mix using RevertAid First Strand cDNA Synthesis kit (Thermo scientific). Quantitative PCR was performed using TB Green Premix Ex Taq II (Takara, Kyoto, Japan). Primer sequences are listed in Table 1. The cyclic conditions consisted of an initial enzyme activation at 95 °C for 5 min followed by 40 cycles of denaturation at 95 °C for 20 s, annealing, and extension including detection of TB Green bound to PCR product at 56 °C for 40 sec. Glyceraldehyde 3-phosphate dehydrogenase (GAPDH) was used as an internal control for normalization. The relative quantities of PCR fragments were calculated using the comparative CT method.

b) Total protein extraction and Western blot analysis.

Hippocampal protein was extracted and expression levels were assessed using Western blotting. After dissecting the hippocampus, the tissue was washed two times with cold Tris-buffered saline (TBS; 20-mM Trizma base and 137 mM NaCl, pH 7.5). Immediately after washing, tissue was lysed with SDS lysis buffer (62.5 mM Trizma base, 2 % w/v SDS, 10 % glycerol) containing 0.1 mM Na3VO4, 3 mg/ml aprotinin, and 20 mM NaF. After brief sonication to shear DNA and reduce viscosity, protein concentration was determined with a detergent-compatible protein assay reagent (Bio-Rad Laboratories) using bovine serum albumin as the standard. After adding dithiothreitol (5 mM) and bromophenol blue (0.1 % w/v), the proteins were boiled, separated by electrophoresis in 10 % polyacrylamide gels (Invitrogen), and transferred onto a polyvinylidene difluoride (PVDF) membrane (Bio-Rad Laboratories). Membranes were blocked on a shaker for 1 h at room temperature. Blocking buffer consisted of TBST (Tris-buffered saline/0.1 % Tween-20) and 5 % skim milk.

Primary antibodies were dissolved in the blocking buffer and the membranes were immunoblotted with antibodies against glucocorticoid receptor (GR; 1:1000, Santa cruz, Sc-1004) and beta-actin (1:1000, Cell Signaling, 4970) for overnight at 4°C. The membranes were incubated in the anti-rabbit (1:1000, Cell Signaling, 7074) dissolved in the blocking buffer at a room temperature for 80 min. The membranes were visualized with ECL-plus solution (Amersham Pharmacia Biotech). Then, the membranes were then exposed to chemiluminescence (LAS- 4000, Fujifilm) for detection of light emission. Western blot results were quantified using ImageJ 1.51 software (National Institutes of Health, Bethesda, MD) after densitometric scanning of the films.

c) Immunohistochemistry.

For perfusion, mice were sacrificed after ANGII infusion by implantation of osmotic pump for 21 days followed by series of behavioral test. All mice were first deeply anesthetized with pentobarbital (100 mg/ kg, i.p.), and perfused through the heart with physiological saline followed with ice-cold phosphate-buffered 4% paraformaldehyde (pH 7.4). Whole brain was dissected and post-fixed in the same fixative for 4 hr at 4°C. Then the brain blocks were cryoprotected in 30% sucrose for 24 hr at 4°C. Sections were cut with an electronic cryotome at a thickness of 25 μm. Immunohistochemical staining was performed with the Elite ABC Kit (Vector Laboratories). Sections were first rinsed with 0.1 M bovine serum albumin three times for 10 min each, then pre-incubated in 0.1 M PBS containing 1% bovine serum albumin and 0.2% Triton X-100 for 30 min. After rinsing twice with 0.1 M PBS containing 0.5% BSA for 10 - 15 min each, sections were incubated with anti-Iba-1 antibody (1:400; Wako, Cat. # 019-19741) or anti-glial fibrillary acidic protein antibody (GFAP; 1:5000, Abcam, ab7260, MA, USA) diluted with 0.1 M PBS containing 0.5% BSA and 0.05% sodium azide at 4°C. After overnight incubation, sections were rinsed and incubated with biotinylated anti-rabbit IgG secondary antibody (Vector) 1:200 diluted with 0.1 M PBS containing 0.5% BSA for 1 hr at room temperature. After rinsing, the sections were incubated with ABC reagent 1:200 diluted with PBS for 1 hr at room temperature and then rinsed with PBS followed with 0.1 M phosphate buffer. Finally, sections were incubated in SIGMA FAST DAB kit (Sigma) until the desired stain intensity developed. Sections were rinsed with 0.05 mol/L phosphate buffer, and then the sections were dehydrated through graded ethanols, cleared in histoclear (Fisher), and cover slipped using Permount (Fisher). Histological analysis was performed using the following procedure. The number of Iba-1-positive neurons in the hippocampus was counted in three sections for each mouse. Starting from the first section (interaural 2.10 mm, bregma -1.70 mm), counts were taken from three coronal sections at

0.135 mm increments. The number of cells that are immunoreactive to Iba-1 was counted by two blinded observers using a microscope in the same brain area (Nikon). For images of hypothalamus, sections were taken from the level of interaural 3.34 mm, bregma – 0.46 mm to the level of interaural 1.50 mm, bregma – 2.30 mm. For images of amygdala, sections were taken from the level of interaural 2.74 mm, bregma – 1.06 mm to the level of interaural 2.22 mm, bregma – 1.58 mm.

d) Determination of serum chemistries.

The serum was isolated and stored at −80 °C until assayed. Serum cytokine (TNF-α, IL-1β, IL-6, IL-17, IL-4, IL-10 and IFN-γ) levels were measured using Bio-Rad Bio-Plex assay (Bio-Rad, Hercules, CA). The cytokine levels were measured and analyzed according to the manufacturer's instruction. Serum corticosterone level was determined by corticosterone EIA kit according to manufacturer's instructions (Cayman Chemical, Ann Arbor, MI).

e) Quantification of serotonin (5-hydroxytryptamine, 5-HT) and its metabolite, 5- hydroxyindoleacetic acid (5-HIAA) by high pressure liquid chromatography and electrochemical detection (HPLC-ECD).

Mice were sacrificed after ANGII infusion by implantation of osmotic pump for 21 days followed by series of behavioral test. Brain tissue blocks were rapidly dissected in ice and homogenized with ice-cold 0.4M perchloric acid, then incubated on ice for 1 hr. After centrifugation at 21,130 g for 30 min at 4°C, the supernatant was filtered using an appropriate column (#Sc1000-1Kt, SigmaPrep spin column). Filtered supernatants were directly injected onto the Nova-Pak C18 reversed-phase column. The mobile phase consisted of 0.1M sodium phosphate monobasic, 0.1mM EDTA, 1mM sodium octyl sulfate, 0.003% trimethylamine, and 10% methanol at pH 3.7. The external standard solutions for each analyte were 5-hydroxytryptamine (5-HT; sc-298707, Santa Cruz) and 5-hydroxyindole-3-acetic acid (5-HIAA; #H8876, Sigma-Aldrich) solutions in HPLC grade water with 0.4M perchloric acid. The flow rate was kept constant at 1 ml/ min. Chromatographic peak analysis was accompanied by identification of unknown peaks in a sample matched according to retention times.

f) Immunofluorescence.

All mice were first deeply anesthetized with pentobarbital (100 mg/ kg, i.p.), and perfused through the heart with physiological saline followed with ice-cold phosphate-buffered 4% paraformaldehyde (pH 7.4). Whole brain was dissected and post-fixed in the same fixative for 4 hr at 4°C. Then the brain blocks were

cryoprotected in 30% sucrose for 24 hr at 4°C. Sections were cut with an electronic cryotome at a thickness of 25 μm. Sections were first rinsed with 0.1 M bovine serum albumin three times for 10 min each, then pre-incubated in 0.1 M PBS containing 1% bovine serum albumin and 0.2% Triton X-100 for 30 min. After rinsing twice with 0.1 M PBS containing 0.5% BSA for 10 - 15 min each, sections were incubated with anti-Iba-1 antibody (1:400; Wako, Cat. # 019-19741) and anti-CD68 antibody (1:200; Abcam, Cat. #ab201340) diluted with 0.1 M PBS containing 0.5% BSA at 4°C. After overnight incubation, sections were rinsed and incubated with Alexa 488-(Abcam, #ab150073, MA, USA) or Alexa-594-(Invitrogen, #A-21203, CA, USA) conjugated secondary antibodies were used for detection. Nuclei were counterstained with 4′6-diamidino-2-phenylindole (DAPI; Sigma, MO, USA). Cells without addition of primary antibody were served as negative controls. Fluorescent images were taken with a confocal microscope (TCSSP5 II, Leica microsystems, Wetzlar, Germany).

Supplementary figures

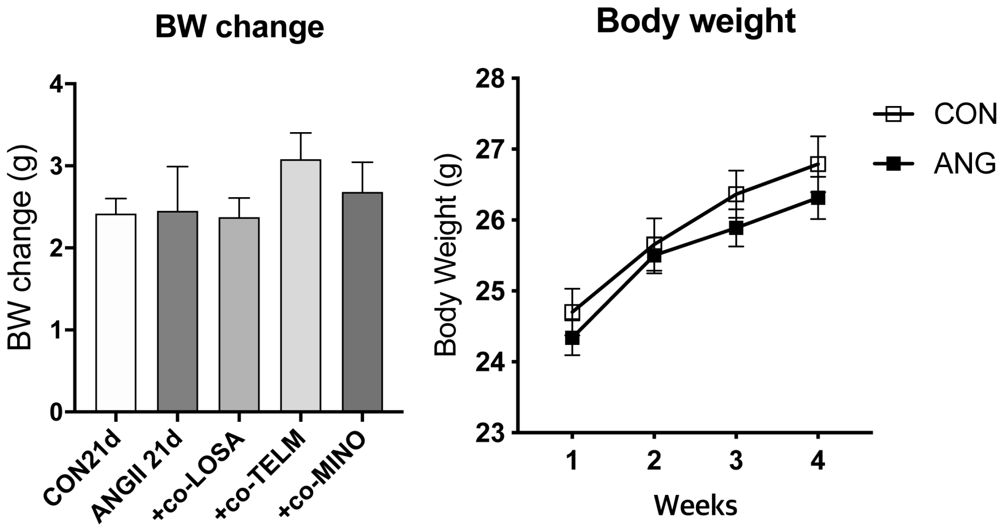

Supplementary Fig. a) The changes in body weight (BW) of mice during 21 days of ANGII infusion were calculated by subtracting initial body weight from the body weight of 21 days and presented as bar graph (left) and body weight measured at the first day of each week for 4 weeks days were shown as line graph (right). n= 30 – 32 in each group. Data shown are mean ± SEM.

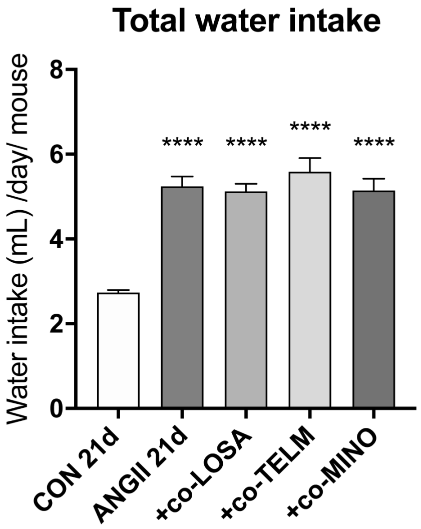

Supplementary Fig. b) The water intakes of mice were measured and shown as water intake (mL)/ day/ mouse. Data shown are mean ± SEM. **** $p < 0.0001$ compared to CON 21d.

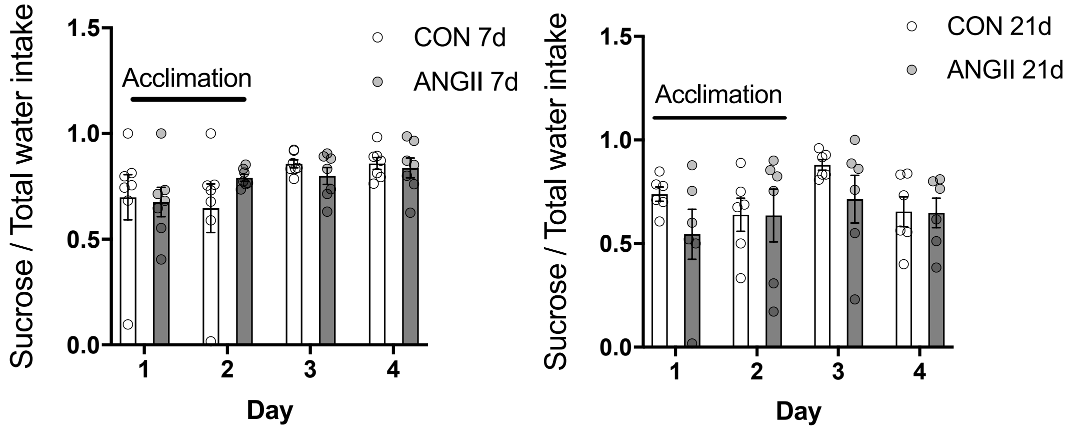

Supplementary Fig. c) Mice were infused with ANGII for 7 days (subacute; 7d)/ 21 days (chronic; 21d) and their sucrose perference was assessed. n = 6 - 7(2 - 5 mice per cage) in each group.

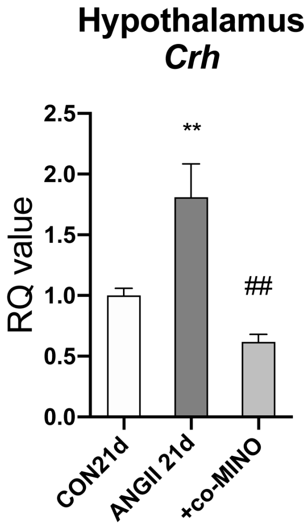

Supplementary Fig. d) The expression of *Crh* (corticotropin-releasing hormone) mRNA in the hypothalamus was assessed by qPCR and the CT values were normalized to controls and RQ values are the ratio of respective transcription factors as a percentage of the controls, n = 7 - 11 in each group. ** $p < 0.01$ compared to CON 21d. ## $p < 0.01$ compared to ANGII 21d.

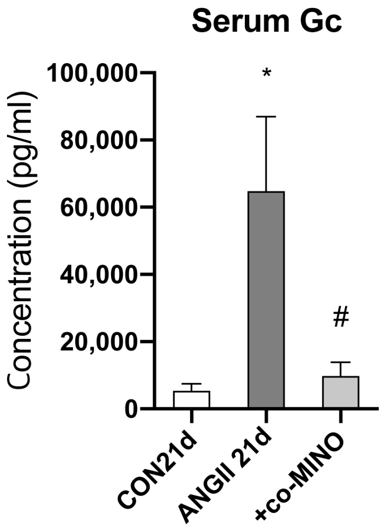

Supplementary Fig. e) Serum glucocorticoid (Gc) levels were measured by an EIA assay and n= 5 in each group. * $p < 0.05$ compared to CON21d. # $p < 0.05$ compared to ANGII 21d.

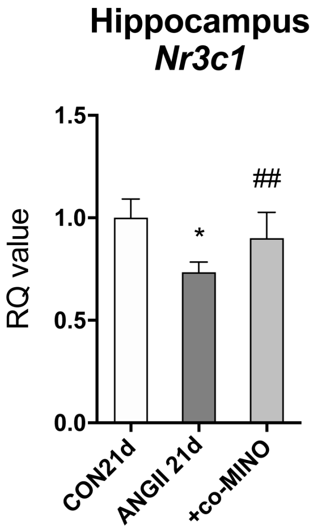

Supplementary Fig. f) The expression of glucocorticoid receptor (*Nr3c1*; GR) mRNA in the hippocampus was assessed by qPCR, and CT values were normalized to controls and RQ values are the ratio of respective transcription factors as a percentage of the controls, n = 7 - 11 in each group. * $p < 0.05$ compared to CON21d. ## $p < 0.01$ compared to ANGII 21d.

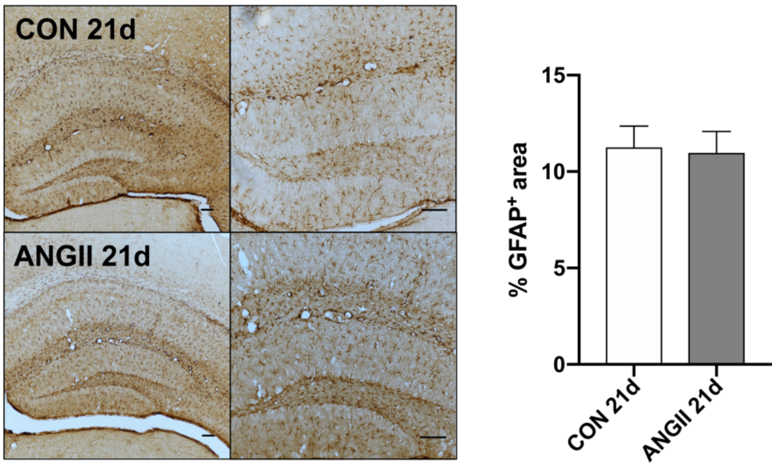

Supplementary Fig. g) An immunohistochemical study was performed to determine GFAP immunoreactivity in the hippocampus of mice, and representative images are shown at 10 X (left) and 20 X (right) magnification. Scale bar = 100 μm. The percentage of area stained by GFAP in the hippocampus were presented by graph, n = 3 in each group.

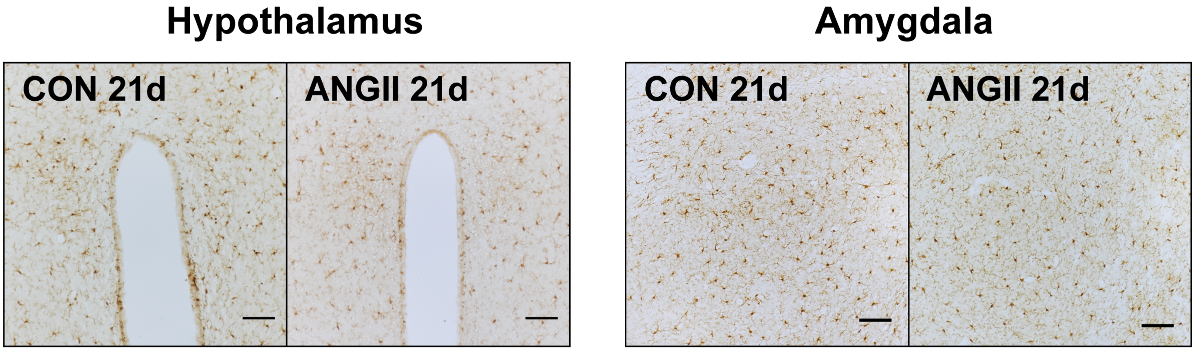

Supplementary Fig. h) An immunohistochemical study was performed to determine Iba-1 immunoreactivity in the hypothalamus (left) and amygdala (right) of mice, and representative images are shown at 20 X magnification. Scale bar = 100 μm.

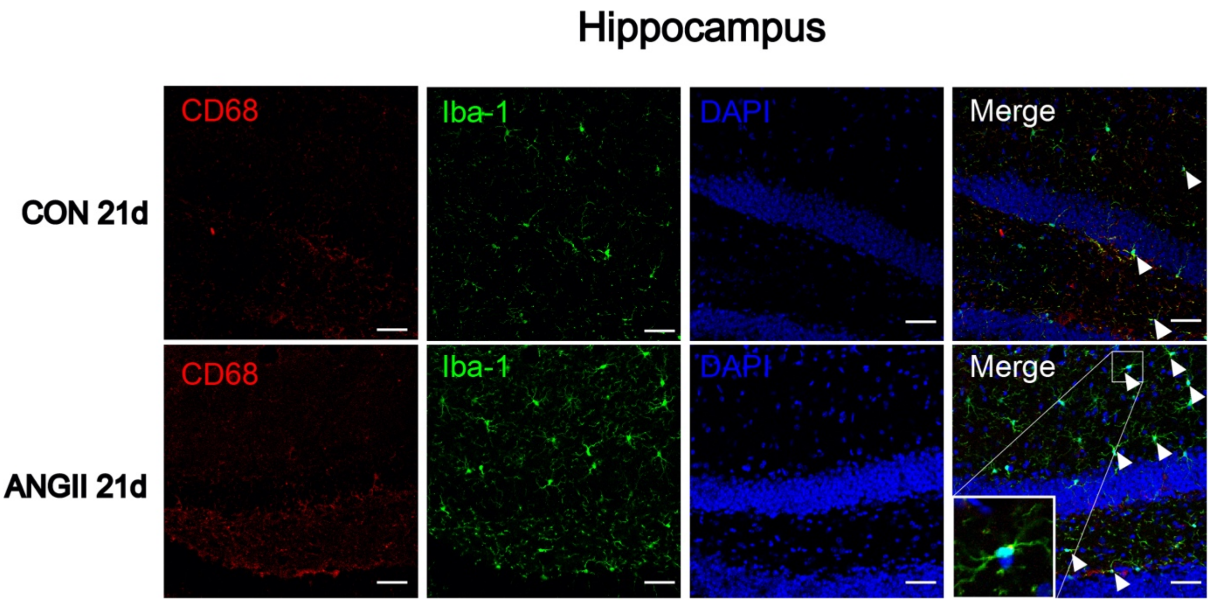

Supplementary Fig. i) The microglial marker Iba-1 and inflammatory microglial marker CD68 were co-stained in the hippocampus of mice. Green, ionized calcium-binding adapter molecule 1 (Iba-1); Red, CD68; Blue, DAPI. Scale bar = 50 μm

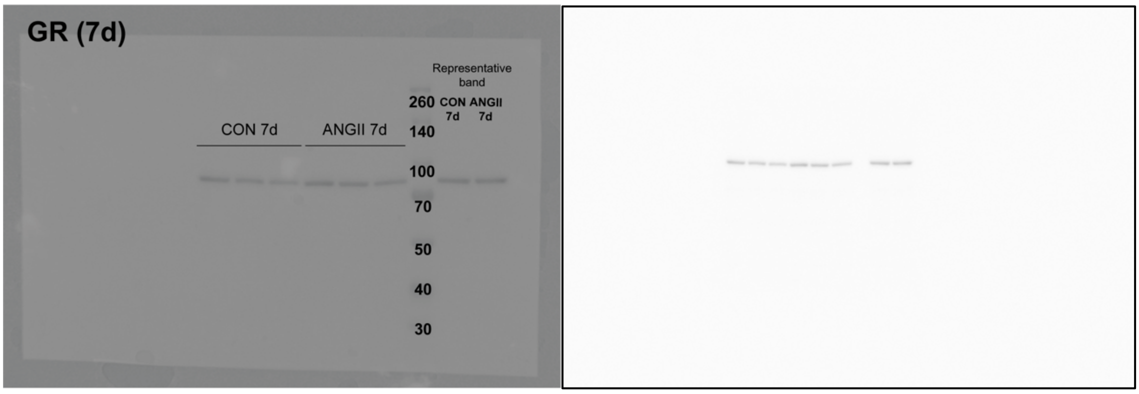

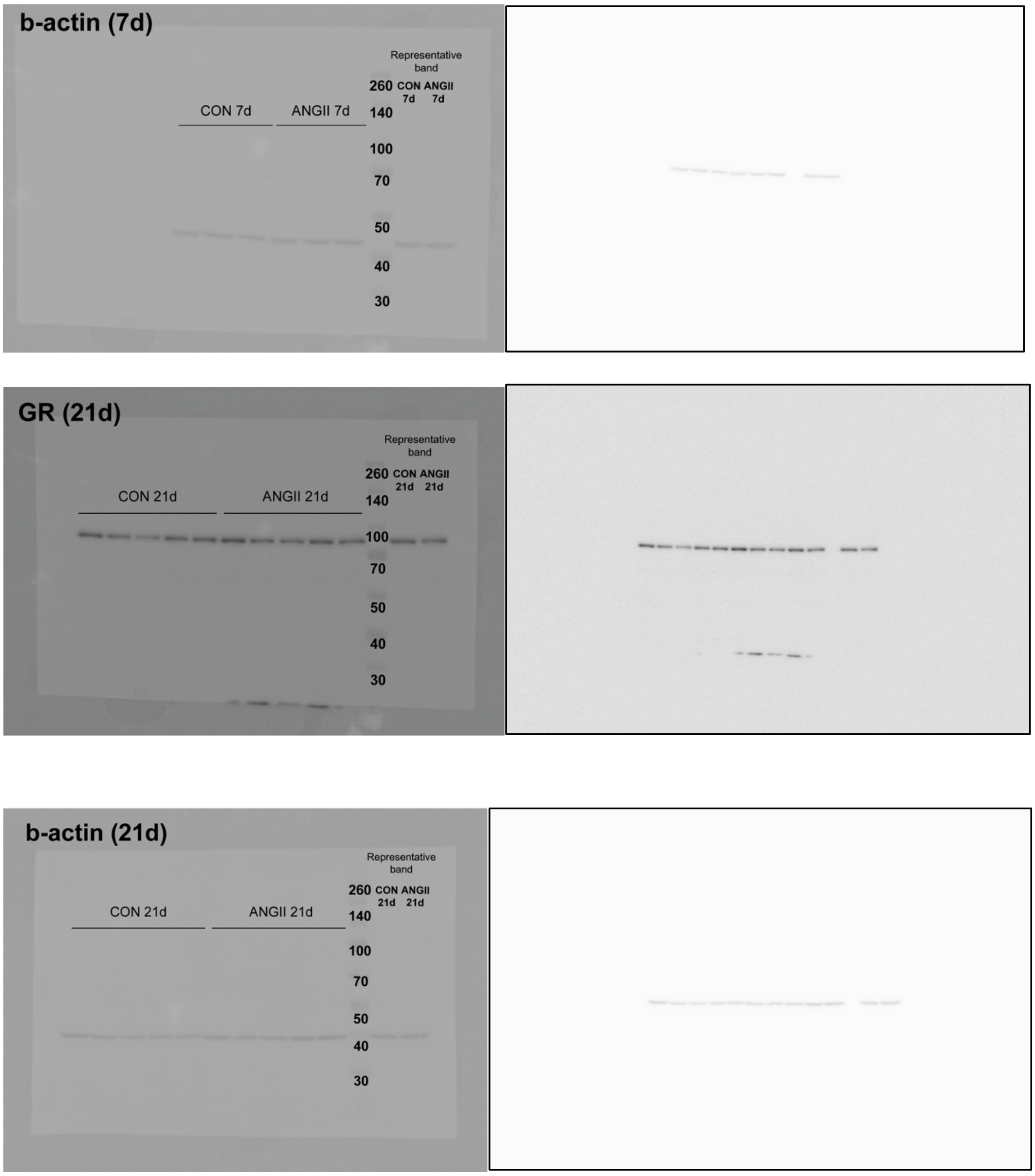

Supplementary Fig. j) Full–length blots of the experiments shown in Fig. 4d. Representative bands are shown in the main text.
